# Comparative analysis of national and foreign players’ performance in Euroleague Basketball

**DOI:** 10.1371/journal.pone.0306240

**Published:** 2024-08-20

**Authors:** Rūtenis Paulauskas, Rokas Kasparavicius, Mykolas Stumbras, Bruno Figueira

**Affiliations:** 1 Educational Research Institute, Education Academy, Vytautas Magnus University, Kaunas, Lithuania; 2 Departamento de Desporto e Saúde, Escola de Saúde e Desenvolvimento Humano, Universidade de Évora, Évora, Portugal; 3 Comprehensive Health Research Centre (CHRC), Universidade de Évora, Évora, Portugal; Universidade da Beira Interior; Research Center in Sports Sciences, Health Sciences and Human Development (CIDESD), PORTUGAL

## Abstract

This study aimed to quantitatively assess the statistical contributions between foreign and national players in men’s Euroleague Basketball. Data from 588 games in the 2021–2022 regular season and 612 games in the 2022–2023 regular season were analyzed through non-participant observation. Paired t-tests and Wilcoxon tests were employed to analyse variables with normal and non-normal distributions, respectively. The analysis indicated significant differences (p<0.05) between local and foreign players across several key variables, including Minutes, Points, Average Points, Usage Percentage, Individual Offensive Rating, Individual Defensive Rating, and True Shooting Percentage. These findings suggest pronounced distinctions between foreign players and national players concerning game volume indicators and game performance efficiency. Foreign players exhibited dominance in critical areas, such as playing time, total points scored, and average points per game, underscoring their substantial contributions to their respective teams. Consequently, these results offer practical implications for players, coaches, and fitness trainers, allowing for the design of more tailored training programs that account for distinct offensive and defensive needs, as well as the heightened physical demands experienced throughout the season.

## Introduction

The contemporary Euroleague Basketball (EB) era started in 2000, and it was the highest-level professional men’s basketball league in Europe to remove restrictions on foreign players’ quotas. Available research up to that moment revealed that quantitative discrepancies between the NBA and EB had diminished and are continuing to reduce, which could have contributed to the tournament being more competitive [1, 2]. It is increasingly difficult to deny that the globalization of sports contributes to the creation of favorable conditions for players and boosts interest in other countries in international sports [3].

The NBA, the most competitive basketball league, has seen a sharp rise in foreign players over the past 20 years, which may have impacted changes in the European and other player markets [4]. Although the migration of athletes can be viewed as positive, there are some issues with it. Due to the tendency for athletes from other nations to be invited to compete effectively in international events, nationality and allegiance are now less significant than athletic prowess and aptitude [3]. According to the squad that best matches their playing style and financial benefits, players frequently switch teams, nations, and continents [5]. Local teams with access to international players could not be motivated to support local athletes, which would be detrimental to the growth of the national sports system. Talent development and identification in professional clubs and national governing institutions are two of the most important topics of discussion currently in adolescent team sports, since they establish the groundwork for future success and performance in senior sports [6, 7]. Due to the numerous subjective variables that scouts and coaches use to choose one player over another, team player recruitment has received critical attention [7]. Why local talent does not satisfy the needs of local teams is an understudied and important subject, with the primary goal of determining the true impact of domestic players on EB teams.

Recent innovations in EB have increased the necessity for objective variables to be evaluated through performance analysis. Understanding basketball players’ performance profiles is critical for clubs looking to optimize player scouting and recruitment in the local and worldwide markets. Previous research has focused on game-related statistics in an attempt to understand what variables are important for effective performance [8]. In fact, there have been recently some attempts to determine which variables contribute the most to differences in game starters and nonstarters [9], on factors that differentiate winning and losing teams [10, 11], the impact of rule changes [12], and the effect of home advantage in basketball games [13, 14]. For example, Ozmen [15] directly compute the marginal contribution and found that the most significant effect on the game was related to turnovers. Yu et al. [16], identified the most relevant technical performance variables, including points per game, field goals made, rebounds, assists, turnovers, blocks, fouls, and steals. Metulini & Le Carre [17] also calculated shot-scoring probabilities in relation to a set of game covariates related to game pressure. Despite the existing statistical links, the data’s rationale is somewhat contentious due to the players’ psychological, physical, and anticipatory skills [18]. Therefore, performance indicators remain more as an outcome of the game which is specific of a group or population. Furthermore, no research has been identified that assessed what contributions from domestic and international players lead to a better outcome in the EB. Recent research explored the differences in game-related statistics between national and foreign female basketball players in the women’s EB, according to playing positions and team ability [19]. The findings revealed that foreign athletes exhibited superior performance in metrics associated with offensive plays, while their national counterparts excelled in indicators linked to defensive actions. This phenomenon parallels certain observations in men’s basketball, where foreign players have consistently outperformed domestic athletes across multiple seasons within the Turkish Basketball League [15]. The examination of technical and physical distinctions between domestic and international soccer players was also investigated and categorized based on playing positions within the China Super League [20]. However, it is essential to note that these analyses in both basketball and soccer may not provide a comprehensive understanding. This limitation arises because these studies were conducted in leagues where tournament regulations-imposed restrictions on the number of foreign players. The constraint of foreign player limits can exert a significant influence, influencing not only the recruitment of high-caliber international athletes but also the promotion of local talents by affording them increased playing opportunities. In the context of football, specifically the UEFA Champions League, where there are no restrictions on player affiliations, it has been observed that teams composed of foreign players exhibit a home advantage effect in terms of goal scoring, while domestic players demonstrate a comparable goal-scoring performance both at home and away venues [21]. These studies thus far have shed light on the influence of including both national and foreign players on the overall performance of sports teams. However, to the best of our knowledge, there exists no prior research that quantifies the statistical contributions of foreign and domestic players in men’s EB. Beginning with the 2016–2017 season, EB adopted a new format, wherein all 18 teams participate in 34 regular season games. A more comprehensive analysis and discussion are warranted, as they could aid team managers, coaches, scouts, and performance analysts in more effectively evaluating the contributions of domestic players within the context of this elite basketball tournament. Consequently, the objective of this study was to examine performance disparities between national and foreign players in EB.

## Materials and methods

### Participants

This study employed a non-participant observational analysis with the objective of comparing two groups of basketball players: national players (NP), who participated in the EB competition representing their own nationality, and foreign players (FP), who competed in the EB while representing a different nation than their own. The dataset included 588 games from the 2021–2022 regular season and 612 games from the 2022–2023 regular season. The uneven number of matches is due to the exclusion of three Russian teams in the 2022 season as a result of the Russian invasion of Ukraine, leaving each of them with 8 remaining regular season games. However, the individual performance metrics of the players were still considered valid during their participation in these games. Within the 18 EB basketball teams, there were 119 NP (59.5% of the total) in the 2021–2022 season and 150 players (60.2% of the total) in the 2022–2023 season. In the 2021–2022 season, there were 200 foreign players (50% of the total), and in the 2022–2023 season, there were 202 foreign players (42% of the total). For the 112 games analysed in this study, which resulted in a total of 2,225 player observations, 1,159 observations were from NP (52.1% of the total), and 1,066 observations were from foreign players (47.9% of the total). Since this study is based on open-access data and does not involve breaches of confidentiality or the use of personally identifiable information, it did not require special ethics committee approval.

### Design

Data concerning player profiles and game-related performance were obtained from the official EB basketball website (https://www.euroleaguebasketball.net/euroleague/) which is consensually considered reliable [18]. To ensure the validity and applicability of this data for our study, we undertook a meticulous process of data verification, including cross-referencing with secondary sources and rigorous data cleaning procedures to correct any discrepancies, enhancing the overall reliability and accuracy of our analysis. The collected variables included match performance (MP) profiles (Table 1) such as minutes (Min), points (Pts), three-point percentage (3P%), two-point percentage (2P%). Calculations on a data set representing advanced basketball statistics were also used as performance indicators. To create our data set, we performed a different calculation using the following equations [22, 23]: percentage of total plays completed (Usage%) = Plays/Team plays, where Plays = Field goals attempted + FT possessions + Turnovers; Offensive rating (Off R) = (points scored/possessions) * 100 where possessions = plays—offensive rebounds, Defensive Rating (Def R) = (points allowed/possessions allowed) * 100 where possessions allowed = plays allowed—offensive rebounds allowed; True Shooting percentage (TS%) = Points / 2* (Field goals attempted + FT possessions); Assist percentage (AST%) = assists/ team field goals attempted + FT possessions, Offensive rebound percentage (ORB%) = Off rebounds/Team off rebounds + Team def rebounds allowed, Defensive rebound percentage (DRB%) = Def rebounds/Team def rebounds + Team off rebounds allowed; Fouls received rate (Fr rate) = fouls received/possessions.

**Table 1:** Performance related variables.

Variable	Operational definitions	Calculations
Minutes	Total number of minutes spent on the court	
TPts	Total number of points scored by one player during the one regular season	
AvgPts	Average number of points per game	AvgPts = Points/Number of games
Usage%	Percentage of total plays completed	Usage% = Plays/Team plays
Ind Off R	Points scored per 100 possessions	Ind Off R = (Points/Possessions) * 100
Ind Def R	Points allowed per 100 possessions	Ind Def R = (Points allowed/Possessions allowed) * 100
TS %	True shooting percentage	TS% = Points / 2* (Field goals attempted + FT possessions)
3P %	3P shooting percentage	3P% = 3P made/3P attempted
2P %	2P shooting percentage	2P% = 2P made/2P attempted
AST%	Assist percentage	AST% = Assists/Team Field goals attempted + FT possessions
ORB%	Offensive rebound percentage	ORB% = Off rebounds/Team off rebounds + Team def rebounds allowed
DRB%	Defensive rebound percentage	DRB% = Def rebounds/Team def rebounds + Team off rebounds allowed
FRv rate	Fouls received rate	FRv rate = Fouls received/Possessions

### Statistical analysis

A descriptive analysis was conducted, providing means and standard deviations for the dataset. Normality assumptions of the data were assessed using the Shapiro-Wilk test. To compare FP and NP, paired t-tests were applied for performance-related variables that followed a normal distribution. For variables that did not meet the normality criteria, the Wilcoxon test was employed. Complementarily, effect sizes (ES) for the variables following a normal distribution were analysed according to Cohen’s d using the following thresholds: small (0.2); medium (0.5); and large (0.8); while the variables that did not show a normal distribution, the ES was calculated by subtracting the average values and the division of the result by the combined standard deviation converted to the following r values: small (0.10); medium (0.30); and (0.50) (large) (Cohen, 1992). The alpha level for all statistical tests was set a priori at α = 0.05 and calculations were carried out using SPSS software (IBM Corp. Released 2016. IBM SPSS Statistics for Windows, Version 24.0. Armonk, NY: IBM Corp).

## Results

The results of match performance are presented in Table 2. We found differences between local and foreign players in the following variables: Minutes (p = <0.001, ES = -0.402), Points (p = <0.001, ES = -0.743), Avg. Points (p = <0.001, ES = -0.915), Usage% (p = <0.001, ES = -0.402), Ind Off R (p = <0.001, ES = -0.327), Ind Def R (p = 0.016, ES = 0.203), TS% (p = 0.032, ES = -0.138).

**Table 2:** Comparison of match performance between national and foreign players.

						95% CI for Cohens’s d	
Variables	Local	Foreign	t	p	Cohen’s d	Lower	Upper
Minutes	279.25±254.90	475.52±261.35	-9.016	<0.001*	-0.758	-0.936	-0.579
TPts	106.50±112.06	196.60±126.49	-8.605^a^	<0.001*	-0.743	-0.926	-0.559
AvgPts	4.38±3.48	7.69±3.71	-10.59	<0.001*	-0.915	-1.104	-0.724
Usage%	0.18±0.07	0.20±0.05	-4.670^a^	<0.001*	-0.402	-0.575	-0.229
Ind Off R	103.32±32.40	111.64±20.00	-3.792^a^	<0.001*	-0.327	-0.499	-0.155
Ind Def R	115.52±24.24	111.99±10.79	2.411^a^	0.016*	0.203	0.037	0.369
TS %	0.56±0.14	0.58±0.09	-2.147^a^	0.032*	-0.188	-0.362	-0.015
3P %	0.37±0.13	0.37±0.12	0.604	0.546	0.058	-0.131	0.248
2P %	0.53±0.16	0.53±0.12	-0.104^a^	0.917	-0.009	-0.184	0.1656
AST%	0.06±0.16	0.06±0.04	-0.986	0.325	-0.088	-0.265	0.088
ORB%	0.06±0.05	0.06±0.04	0.352	0.725	0.032	-0.147	0.211
DRB%	0.29±0.18	0.29±0.12	-0.824	0.411	-0.073	-0.247	0.101
FRv rate	0.13±0.06	0.13±0.05	-0.301^a^	0.763	-0.027	-0.202	0.149

Notes: TPts = Total number of points; AvgPts = Average points; Ind Off R = Individual Offensive Rating; Ind Def R = Individual Defensive Rating; TS% = True shooting percentage; 3P% = three-point percentage; 2P% = two-point percentage; AST% = Assist percentage; ORB% = Offensive rebound percentage; DRB% = Defensive rebound percentage; FRv rate = Fouls received rate.

* indicates significant differences

^a^Levene’s test is significant (p<0.05), suggesting a violation of the equal variance assumption.

## Discussion

The primary aim of this study was to investigate performance disparities between NP and FP in EB. To the best knowledge, there are no studies examinating the player performance in EB based on advanced statistical analysis, as illustrated in Table 2. Our analysis suggests significant contrast in playing time between NP and FP, substantiating the presence of differences. This bias becomes evident through the unequal distribution of playing time within the preeminent European tournament. As Poli & Besson [24] have argued, the international migration of athletes has emerged as a noteworthy aspect of globalization within the sports industry. The allocation of minutes on the court further accentuates the profound influence that FP exert on the competitiveness of EB clubs. Concurrently, this raises a conspicuous concern for FP: the physical workload they endure is nearly twice that of their local counterparts.

During a single regular season in European EB, our analysis indicates a notable disparity (p < 0.001) in the accumulation of TPts scored by individual players, with FP showing significantly superior scoring performance when compared to their NP counterparts. Previous research in the realm of basketball player success has primarily emphasized scoring abilities, often regarded as a pivotal component in player profiling [25, 26]. While previous studies have often focused on scoring abilities as key to basketball player success, our findings suggest a correlation between player origin and scoring, with FPs typically achieving higher total and average point values.

Prior studies have already established the substantial influence of FP on scoring within national competitions [9, 27]. Nevertheless, it’s essential to acknowledge that the EB competition operates at a distinctive level, and the selection of players for this league sets unique standards that may not be universally applicable.

Oliver’s [23] Ind Off R quantifies the points a player has scored in a hypothetical 100 possessions. This statistic, while not indicative of the pace of play, serves as a quantitative measure that effectively distinguishes between FP and NP, with a significance level of p < 0.001. Furthermore, it sheds light on the offensive creativity displayed by the players, as highlighted by Memmert [28]. It’s important to note that Ind Off R is not tailored exclusively to taller players and holds greater relevance for proficient three-point shooters. Nonetheless, these statistics enable us to characterize FP as skilled long-range shooters, even within the sample group that includes taller players, who typically operate closer to the basket.

The purpose of calculating the Ind Def R is to assess a player’s ability to prevent their opponent from scoring individually. Our collected data reveals that FP allow opponents to score fewer points compared to NP, with a significance level of p<0.016. This finding contradicts earlier studies, which have suggested that foreign players are typically recruited for offensive roles, while national players are predominantly utilized for defensive purposes [27]. Furthermore, our research aligns with previous studies indicating that achieving high offensive and defensive ratings in a regular season is an uncommon occurrence among basketball players [8, 29]. Our study distinguishes foreign players in the Euroleague tournament as characterized by versatility, exhibiting both offensive and defensive proficiency. Recent research further supports the notion that the EB tournament emphasizes player versatility, a quality that has long been esteemed in the NBA [1, 2].

### Efficiency vs volume

When evaluating the entirety of the data, particularly in basketball, the importance of quantity often surpasses that of efficiency, as a higher-scoring output generally equates to a greater advantage. However, in player analysis, particularly when considering season-long data, the emphasis on efficiency becomes more pronounced. One of the key efficiency metrics is Usage %, a parameter that indicates the percentage of a team’s plays in which a player actively participated, including making scoring attempts, free throw attempts, or turnovers [23]. Our study has identified a significant difference in Usage % between FP and NP. This distinction underscores the distinct roles played by FP and NP in their team’s offensive strategies and quantifies the opportunities they have to contribute to scoring. Given the inherent dynamics of basketball with five players on the court, it’s inevitable that some players will be more involved offensively than others. Thus, a player’s Usage % serves as a valuable indicator of the leadership role FP assume in their team’s on-court performance. Cluster analysis conducted in the context of the Chinese Basketball Association (CBA) has similarly shown a significantly higher Usage% among foreign players compared to their local counterparts, particularly highlighting the advantage of three-point shooters [27]. The true value of this metric lies in its ability to provide context to other statistics [22]. Higher Usage % players, in essence, have more opportunities to influence the game. However, it is important to note that there exists a negative relationship between Usage % and scoring efficiency, as established by Memmert [28]. This suggests that players with both high scoring efficiency and a high Usage % are a rare find. Nevertheless, a thorough analysis of match performance demonstrates that despite having a higher Usage%, FP are characterized by superior offensive efficiency.

Another crucial criterion for evaluating scoring efficiency is the individual TS%, where FP exhibited a notable advantage. The TS%, incorporates free throws, 2-pointers, and 3-pointers, providing a comprehensive assessment of the overall value of these scoring methods [22, 30]. Our analysis seems to show a significant advantage for FP in this aspect (p = 0.032). This advanced statistical metric accounts for all scoring elements and stands as the most comprehensive measure of shooting proficiency. It is alternatively referred to as adjusted shooting percentage, effective shooting percentage, efficiency percentage, points per attempt, and field goal efficiency [28]. Although we observed that 2P% and 3P% do not exhibit significant differences between the two player groups, the substantial variation in TS% may be attributed to variations in free throw possessions.

While the number of assists is a valuable parameter for evaluating player performance, AST % is an advanced statistical metric that offers a more nuanced assessment of a player’s passing skills [22]. AST% draws on two key components: the percentage of assists derived from team field goals attempted and free throw possessions. Because AST% is independent of the effects of pace and overall volume, it provides a more precise indication of a player’s efficiency in generating assists per team possession. Essentially, this statistic quantifies what percentage of their teammates’ field goals a player assists when they are on the court [23]. In our analysis, the AST% values for the two player groups were not significantly different. However, considering that FP possess a substantial advantage in terms of volume-related characteristics, the equivalency in AST% between FP and NP can be regarded as a noteworthy contribution. It is important to recognize that passing proficiency is not solely dependent on the technical abilities of players; it is also intricately linked to their decision-making skills and perceptual abilities [9]. These factors are associated with a player’s maturity, their capacity to read the defence, and their understanding of when and where to deliver the ball to a teammate [29, 31].

ORB% is a metric that quantifies the percentage of potential rebounds secured by a player while on the court. In practical terms, both groups exhibit similar proficiency in gathering rebounds following missed shots by their teammates, with no significant distinction between the two groups. A similar pattern is evident in the context of DRB%, where the distribution of rebound efficiency remains consistent for FP and NP. This finding contradicts previous assertions suggesting that FP are primarily recruited for offensive roles, while NPs typically assume defensive, rebounding, and assisting responsibilities on the court [19]. FRv, which serves as a defensive indicator, further corroborates this trend by highlighting the absence of significant disparities between the two player groups.

### Practical applications

By leveraging the in-depth analysis from our recent study, basketball teams have the opportunity to refine their talent scouting strategies, with a particular focus on the distinctive strengths that foreign national players contribute to the game. This approach, when integrated with customized training programs derived from the data, has the potential to significantly enhance the performance of individual players and improve overall team dynamics, ultimately leading to more strategic and impactful gameplay.

## Conclusion

Our study provides an in-depth analysis of the distinct performance metrics of foreign and national players in Euroleague Basketball, revealing that foreign players often excel in key areas such as playing time, total points, and average points per game. This challenges the traditional perception of foreign players being mainly offensive, highlighting their diverse contributions. However, it’s important to recognize that these insights are specific to the Euroleague context and may not extend to other basketball leagues with different standards and styles of play. Therefore, while our findings offer valuable considerations for team scouts and player managers in the Euroleague, we caution against a broad application of these results to other settings.

### Limitations

This study, primarily based on quantitative analysis, acknowledges its limitations in capturing the full spectrum of player attributes, including mental, social, and psychological aspects. As these factors are integral but not fully represented in the metrics used, our findings should be interpreted with caution, especially regarding their broader applicability. Future research should aim to include a more diverse range of player attributes and intra-player variability, to enhance understanding in this field. Also, It is important to note that, due to the retrospective nature of this study, the present findings should be interpreted as correlations and not causations.
